# Effect of IVIG Formulation on IgG Binding to Self- and Exo- Antigens *In Vitro* and *In Vivo*

**DOI:** 10.1371/journal.pone.0161826

**Published:** 2016-08-25

**Authors:** Susann Cattepoel, Annette Gaida, Alain Kropf, Marc W. Nolte, Reinhard Bolli, Sylvia M. Miescher

**Affiliations:** 1 CSL Behring AG, Bern, Switzerland; 2 CSL Behring GmbH, Marburg, Germany; Charite Universitatsmedizin Berlin, GERMANY

## Abstract

In relation to the recent trials of Intravenous Immunoglobulin (IVIG) in Alzheimer’s Disease (AD) it was demonstrated that different IgG preparations contain varying amounts of natural anti-amyloid β (Aβ) antibodies as measured by ELISA. We therefore investigated the relevance of ELISA data for measuring low-affinity antibodies, such as anti-Aβ. We analysed the binding of different commercial Immunoglobulin G (IgG) preparations to Aβ, actin and tetanus toxoid in different binding assays to further investigate the possible cause for observed differences in binding to Aβ and actin between different IgG preparations. We show that the differences of commercial IgG preparations in binding to Aβ and actin in ELISA assays are artefactual and only evident in *in vitro* binding assays. In functional assays and *in vivo* animal studies the different IVIG preparations exhibited very similar potency. ELISA data alone are not appropriate to analyse and rank the binding capacity of low-affinity antibodies to Aβ or other endogenous self-antigens contained in IgG preparations. Additional analytical methods should be adopted to complement ELISA data.

## Introduction

Patients with immune defects such as agammaglobulinaemia or hypogammaglobulinaemia rely on substitution therapy using Intravenous immunoglobulin (IVIG) preparations. IVIG is a fractionated blood product produced from thousands of pooled blood donations. These products therefore contain the accumulated human antibody repertoire, which consists not only of antibodies against exo-antigens such as Tetanus Toxoid and Varizella Zoster Virus (VZV) but also antibodies against abundant self-antigens such as the amyloid-beta peptide (Aβ) and actin [1–3]. Antibodies against tetanus toxoid, which are present in the plasma at high concentration due to extensive vaccination of the donors, have a high affinity to the antigen. Similarly, infection with VZV generates high-titer, high-affinity antibodies, whereas Aβ as well as actin generate low-titer, low-affinity antibodies. These so-called natural antibodies (nAbs) arise against self-antigens in the absence of vaccination or passive-immunization, have not undergone affinity maturation and are therefore low-affinity and poly-reactive. NAbs have important functions in tissue homeostasis, tumor surveillance and immune modulation [4].

Due to the well documented anti-inflammatory and immuno-modulatory effects of IVIG preparations, a large number of diseases with an autoimmune pathophysiology have also been treated with IVIG [5]. In 1982, IVIG was introduced for the treatment of autoimmune diseases such as multiple sclerosis, myasthenia gravis, chronic inflammatory polyneuropathies and Guillain-Barré syndrome [6–8]. There are reports that IVIG may also be effective for the treatment of Alzheimer’s disease (AD) and other neurodegenerative disorders [9, 10]. Recent papers reported that serum from healthy elderly individuals had higher levels of natural anti-Aβ antibodies than did serum from AD patients of similar age [9, 11–13]. Therefore, it was hypothesized that the beneficial effects of IVIG in patients with AD might be related to their higher titer of anti-Aβ antibodies after treatment compared to the lower titer of anti-Aβ antibodies in serum from untreated AD patients [14]. Conversely, subsequent studies reported equal or increased titers of circulating anti-Aβ antibodies in untreated AD patients based on ELISAs performed on immunoglobulin preparations treated at low pH to dissociate antigen from antibody [11]. However, it has been reported that exposure of low -affinity polyclonal IgG to low pH may result in structural changes or even partial denaturation of antibodies which may generate artefactual increases in apparent activity against endogenous antigens [15, 16]. *In vitro* assays such as ELISAs employing IgG isolated from human plasma using methods that require a treatment at low pH may therefore artefactually under- or over- estimate IVIG activity against endogenous antigens [17–19].

In conventional ELISAs we and others [11, 13] found that IVIG preparations display differences in binding to Aβ. In this study we also analyzed the binding to actin, a second low-affinity self-antigen and found comparable differences between commercially available IgG preparations but not to tetanus toxoid or VZV, examples of exo-antigens generating high affinity antibodies. Due to these potential causes of inaccuracy, we decided to investigate the possible cause for the observed differences in binding to Aβ and actin between different IgG preparations using improved ELISA protocols [15].

## Materials and Methods

For this study 7–9 week old female Clr:CD(SD) rats were intravenously injected with IVIG preparations. Blood samples were taken at different time points. Animals were anaesthetized by CO_2_ inhalation during baseline and 2 min blood sampling, whilst 6h (n = 5) and 24h (n = 5) blood samples were taken under deep injection anesthesia (ketamine/xylazine) prior to exsanguination. Animal experiments were approved by the Regierungspräsidium Giessen (local ethics institution) and were performed in compliance to all federal and state regulations.

### Immunoglobulin preparations

The following Immunoglobulin G (IgG) preparations for intravenous use were used: Gamunex 10% (Grifols, Barcelona, Spain), Gammagard Liquid 10% (Baxter, Deerfield, IL, USA), Octagam 10% (Octapharma, Lachen, Switzerland) and Privigen 10% (CSL Behring, Bern, Switzerland).

For further studies IgG was formulated to 250 mM Proline (pH 4.8), 250 mM Glycine (pH 4.7) or 200 mM Glycine (pH 4.2), respectively.

Privigen Fc fragments were prepared from IVIG (Privigen) as described previously [20]. The Privigen Fc fragments were used as a negative control where indicated.

### Preparation of Aβ42

Recombinant Aβ42 peptide was obtained as ultra-pure hexafluoro-2-propanol (HFIP) film from rPeptide (Bogart, GA, USA) and prepared for use as described previously [20].

Aβ oligomers were prepared according to Lambert et al. [21] with some modifications [20]. The oligomers were stabilized by cross-linking with 25 mM peroxynitrite for 20 min at room temperature (RT) [22]. Fibrillar FITC-Aβ42 was prepared as described previously [20].

### ELISA

Peroxinitrite cross-linked Aβ 1–42 oligomers were coated at 0.33 μM in PBS pH 7.4 on 96-well plates (Nunc Maxisorb; Nunc, Penfield, NY, USA) for 1h at 37°C. Human non-muscle actin (Cytoskeleton, Denver, CO, USA) and formalin-treated tetanus toxoid were coated at a concentration of 5 μg/ml in carbonate buffer, pH 9.6 at 4°C o/n. Wells were washed once with Wash Buffer (1x PBS, 0.05% (v/v) Tween-20) and blocked with Smart Block (Candor Bioscience GmbH, Wangen, Germany) for 1 hour at 37°C. After washing, IgG preparations were diluted in Low Cross Buffer (LCB) (Candor Bioscience) and added to the plate. The following control antibodies were used: anti-Aβ monoclonal antibody 6E10 (BioLegend, Dedham, MA, USA) and anti-β-Actin monoclonal antibody (Sigma, St. Louis, MO, USA). As a negative control a mix of human IgG1 (68.0%), IgG2 (28.0%), IgG3 (3.0%), IgG4 (1%) myeloma proteins (The Binding Site, Birmingham, UK) was used. Following incubation and washing the plates were incubated with the following secondary antibodies: rabbit anti-human IgG-HRP (Dako, Glostrup, Denmark) at 0.5 μg/ml and goat anti-mouse IgG-HRP (Dako) at 0.5 μg/ml in antibody buffer (Superblock; Thermo Fisher Scientific, Waltham, MA, USA) in PBS Tween 0.05% (1:5)). Wells were washed 5 times and subsequently developed with ultra-sensitive TMB (Fitzgerald Industries, Acton, MA, USA). The reaction was stopped with 1M sulphuric acid and absorbance was measured at 450nm by EnVision Multilabel Reader (Perkin Elmer, Waltham, MA, USA). The anti-tetanus toxoid Antibody and anti-Varicella Zoster Virus (VZV) Antibody ELISA Kits (The Binding Site) were performed according to the manufacturer’s protocol. Each experiment was performed at least three times.

### Real-Time Binding Assays (Octet)

The Octet assays were performed as described previously [20] and measured in a 96-well format on an Octet QKe device (FortéBio Inc., Menlo Park, CA, USA) [23]. The binding of IgG preparations to unconjugated biosensor tip was subtracted as background. The assays were analyzed and fitted with the Octet Software 7.0.1.1 (FortéBio Inc.). Each experiment was performed at least three times.

### Thioflavin T Aggregation Assay

Aggregation of Aβ was monitored by using the Thioflavin T (ThT) binding assay, which was performed as described previously [20]. The fluorescence intensity was measured in an EnVision multilabel reader (Perkin Elmer). Samples were measured in triplicates and each assay was performed a minimum of 3 times.

### *In Vitro* Cytotoxicity Assay

SH-SY5Y human neuroblastoma cells were obtained from ATCC (ATCC, St. Cloud, MN, USA) and cultured according to protocol. Cells were plated and differentiated with 10 μM all-trans Retinoic acid for 72h at a density of 10,000 cells per well in a 96-well microtiter plate (TPP, Trasadingen, Switzerland). Aβ42 monomers (10 μM final concentration) were added in 100 μl of fresh medium with indicated dilutions of test substances and incubated for 72h. Samples were measured in hexaplicates and each assay was performed a minimum of 3 times. LDH toxicity assay (Sigma) was performed according to the manufacturer’s protocol and measured in an EnVision multilabel reader (Perkin Elmer).

### *In Vitro* Phagocytosis Assay

The phagocytosis assay was performed with the BV-2 microglial cell line (Dr. V. Bocchini, University of Perugia (Perugia, Italy) [24]) according to Webster et al. [25] with some modifications [20]. In addition to Privigen Fc fragment, which does not mediate phagocytosis, Cytochalasin D (CytoD), which blocks receptor-mediated endocytosis, was used as control. Propidium iodide staining was performed to determine the number of dead cells. After incubation the cells were subjected to FACS analysis to determine FITC-fluorescence intensity (BD FACSdiva; BD Biosciences, Franklin Lakes, NJ, USA) and analyzed with FlowJo (Tree Star Inc., Ashland, OR, USA). Each Experiment was performed at least three times.

### *In Vivo* Pharmacokinetic Study

7–9 weeks old female Clr:CD(SD) rats (Charles River Labortories GmbH, Sulzfeld, Germany) were intravenously injected with 500 mg/kg of Privigen 10% (CSL Behring), Octagam 10% (Octapharma), Gammagard 10% (Baxter) or Gamunex 10% (Grifols), respectively (n = 10/group). Blood samples were taken at baseline (before treatment), and 2 min, 6h and 24h after respective injections. Thereby, animals were anaesthetized by CO_2_ inhalation during baseline and 2 min blood sampling, whilst 6h (n = 5) and 24h (n = 5) blood samples were taken under deep injection anesthesia (ketamine/xylazine) prior to exsanguination. Subsequently, samples were processed to plasma (10% citrate) and thereafter analyzed by ELISA methods to measure anti-Aβ, -actin, -tetanus toxoid and -varizella zoster virus (VZV) activity of human IgG. Furthermore, total IgG, proline and glycine levels were determined by Nephelometry and HPLC, respectively. Rats were housed in a humidity- and temperature-controlled room with 12h light/dark cycle and free access to food and water. Throughout the study (e.g. before and after injections and blood sampling), rats were monitored for evidence of ill health and reactions to treatment. Animal experiments were approved by the Regierungspräsidium Giessen (local ethics institution) and were performed in compliance to all federal and state regulations.

### Statistics

Data were expressed as means ± SD. Statistical analysis was performed by Student’s t-test or ANOVA and Bonferroni´s Post-hoc test using GraphPad Prism 5 (GraphPad Software Inc., La Jolla, CA, USA). A value of p < 0.05 was considered statistically significant.

## Results

### Binding of different IgG preparations to oligomeric Aβ

**Fig 1:**
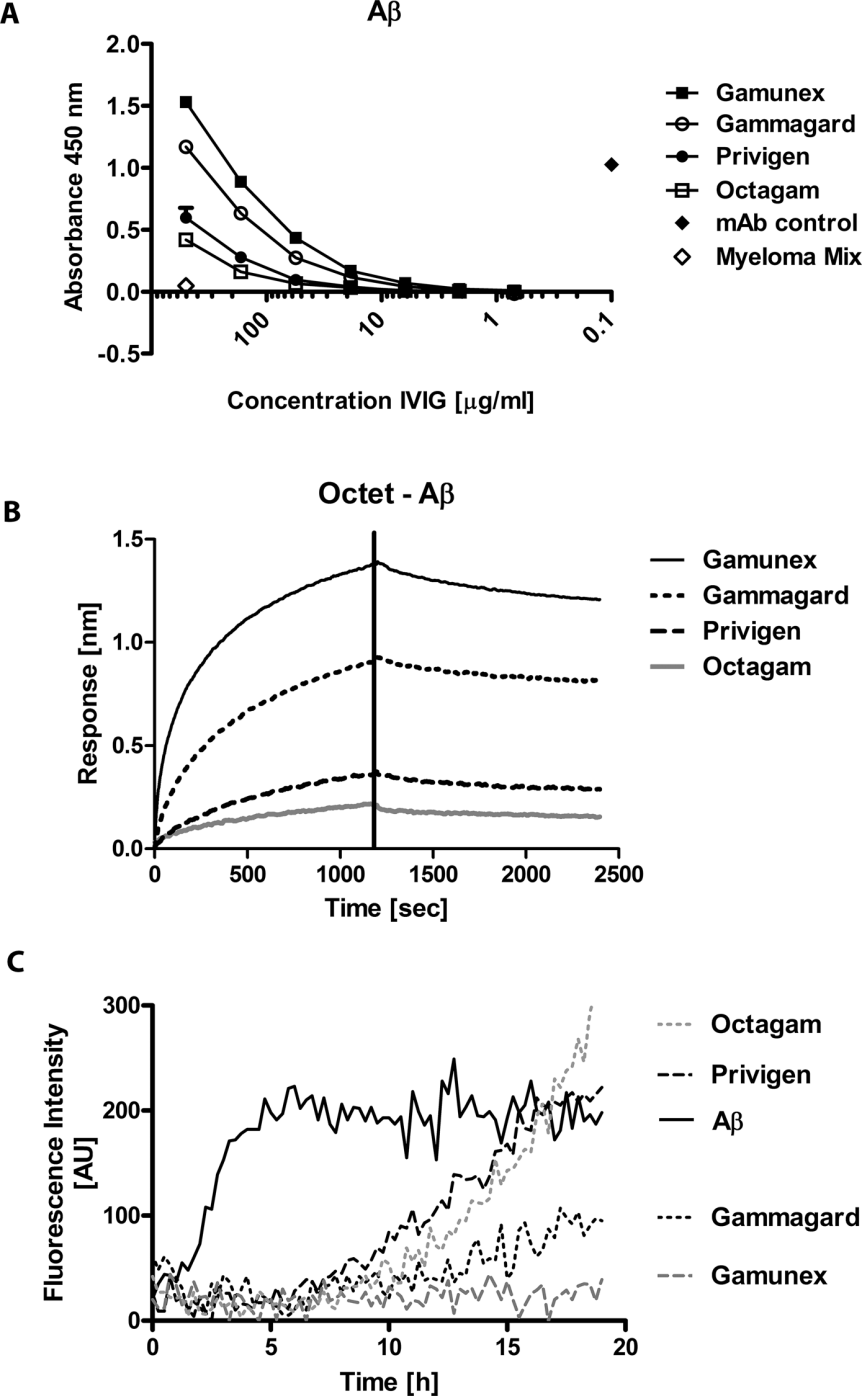
*In vitro* Comparison of commercial IgG preparations activity to Aβ by ELISA, Octet and Thioflavin T. (A) ELISA analysis of different commercial IgG preparations on plate-immobilized Aβ. (B) Octet measurements of the same commercial IgG preparations. (C) Inhibition of Aβ aggregation by different IgG preparations as measured by Thioflavin T fluorescence assay. The panel shows combined results from repeated measurements (n = 3) with different lots.

The binding of the IgG preparations Gamunex (3 lots), Kiovig (3 lots), Octagam (4 lots) and Privigen (4 lots) to oligomeric Aβ42 was measured by ELISA. Fig 1A shows the binding of the different products to plate-bound Aβ42 oligomers. The panel shows combined results from repeated measurements (n = 3) with different lots. The signals generated with Gamunex and Kiovig were consistently higher as compared to Octagam and Privigen, but we did not observe lot-to-lot variations. The same differences were observed when real-time binding was measured in the Octet system using Aβ42-coated biosensor tips (Fig 1B). Furthermore, measurement of Aβ42 aggregation in the Thioflavin T assay revealed that the inhibitory effect of Gamunex and Gammagard on Aβ42 aggregation was stronger compared to Privigen and Octagam (Fig 1C). Next, we explored if the observed differences were specific for only anti-Aβ antibodies or a certain category of antibodies, such as antibodies to self-antigens and investigated a potential influence of the different formulations on the ELISA/Octet signal.

### The effect of formulation is pronounced for IgG binding to self-antigens, but not the exo-antigen Tetanus Toxoid

**Fig 2:**
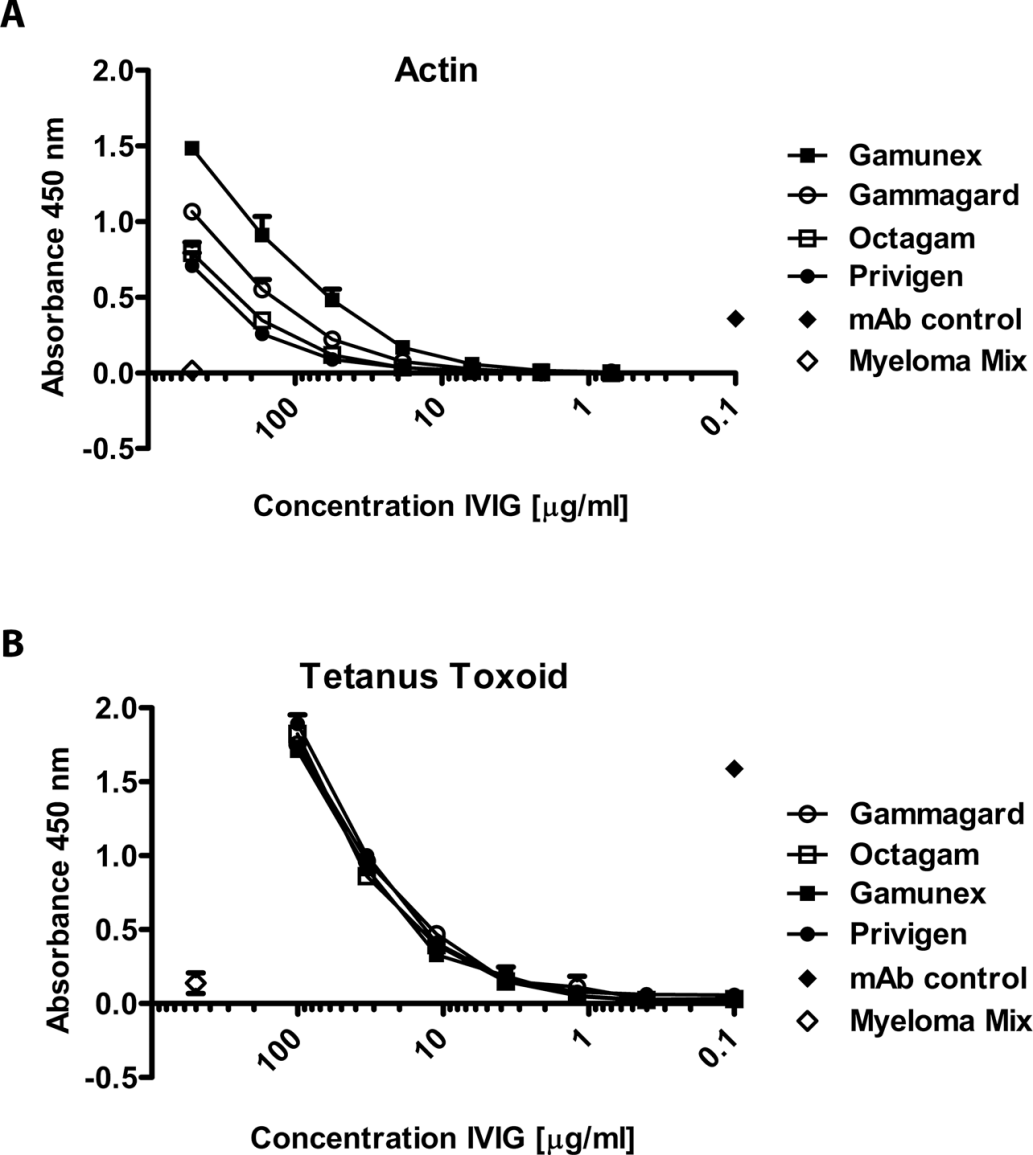
*In vitro* Comparison of commercial IgG preparations activity to actin and tetanus toxoid by ELISA. ELISA measurement on (A) on plate-bound actin and (B)on immobilized tetanus toxoid. The panel shows combined results from repeated measurements (n = 3) with different lots.

Fig 2 shows the binding of the four previously analyzed IgG products to the intracellular self-antigen actin (Fig 2A) and the pathogen-associated exo-toxin of *Clostridium tetani* (Fig 2B) as analyzed by ELISA. The panel shows combined results from repeated measurements (n = 3) with different lots.

The previously observed differences in binding to Aβ42 between IgG products also occurred with the second self-antigen, actin (Fig 2A), but there were no differences in binding to tetanus toxoid (Fig 2B) between the different products. These findings were confirmed by real-time binding to actin and tetanus toxoid using the Octet system (data not shown).

In the next step we focused on the different formulations of the IgG preparations. Privigen is formulated in 250mM L-proline at pH 4.8, Gamunex and Gammagard contain glycine at pH 4.2 and 4.7, respectively. Therefore, an unformulated process intermediate of Privigen was formulated to 250 mM Proline pH 4.8, 250 mM Glycine pH 4.7 or 200 mM Glycine pH 4.2, respectively.

**Fig 3:**
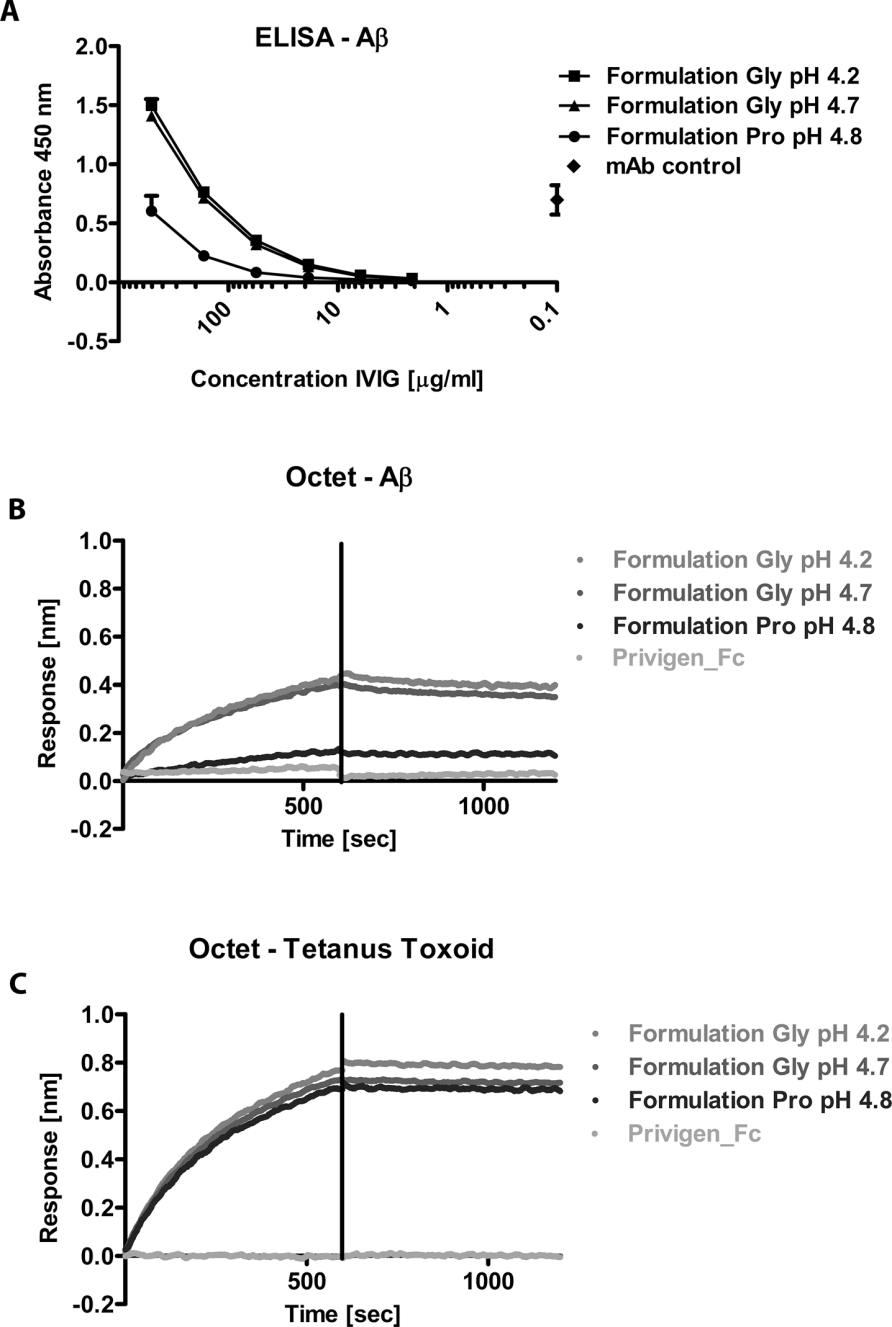
*In vitro* Comparison of reformulated IgG preparations activity to Aβ, actin and tetanus toxoid by ELISA and Octet. Unformulated Privigen (in H_2_O) was formulated in Proline pH 4.8, Glycine pH 4.7 or Glycine pH 4.2. (A) ELISA measurements on plate-bound Aβ oligomers. The panel shows combined results from repeated measurements (n = 3). (B) Octet binding measurements to immobilized Aβ and **C** to bound tetanus toxoid. Privigen Fc fragments were used as negative controls.

Analysis of these differently formulated IgG preparations in Aβ42- and actin- ELISA showed comparable differences in binding to the self-antigens as the originally formulated IgG products. The IgG preparation formulated in Glycine at pH 4.7 and pH 4.2 exhibited a higher signal to Aβ42 (Fig 3A) compared to the preparation formulated in Proline at pH 4.8. No differences in binding to tetanus toxoid between the different formulations were detected by ELISA (data not shown).

Measurements in the Octet system confirmed the results of the ELISA data i.e. differences between the formulations in binding to Aβ42 (Fig 3B) and actin (data not shown) were observed but no differences in binding to tetanus toxoid (Fig 3C). As expected, Privigen Fc, which was used as negative control, generated no signal.

These results indicate that the formulation of the IgG preparation has an impact on *in vitro* binding assays for low- to medium-affinity antibodies (antibodies to self-antigens) but had no influence for high-affinity antibodies (antibodies to exo-antigens). In order to investigate the relevance of the observed differences in *in vitro* binding assays on function and activity of IgG preparations, we performed cell-based *in vitro* functional assays.

### Formulation-dependent differences in binding have no impact on *in vitro* functional assays

**Fig 4:**
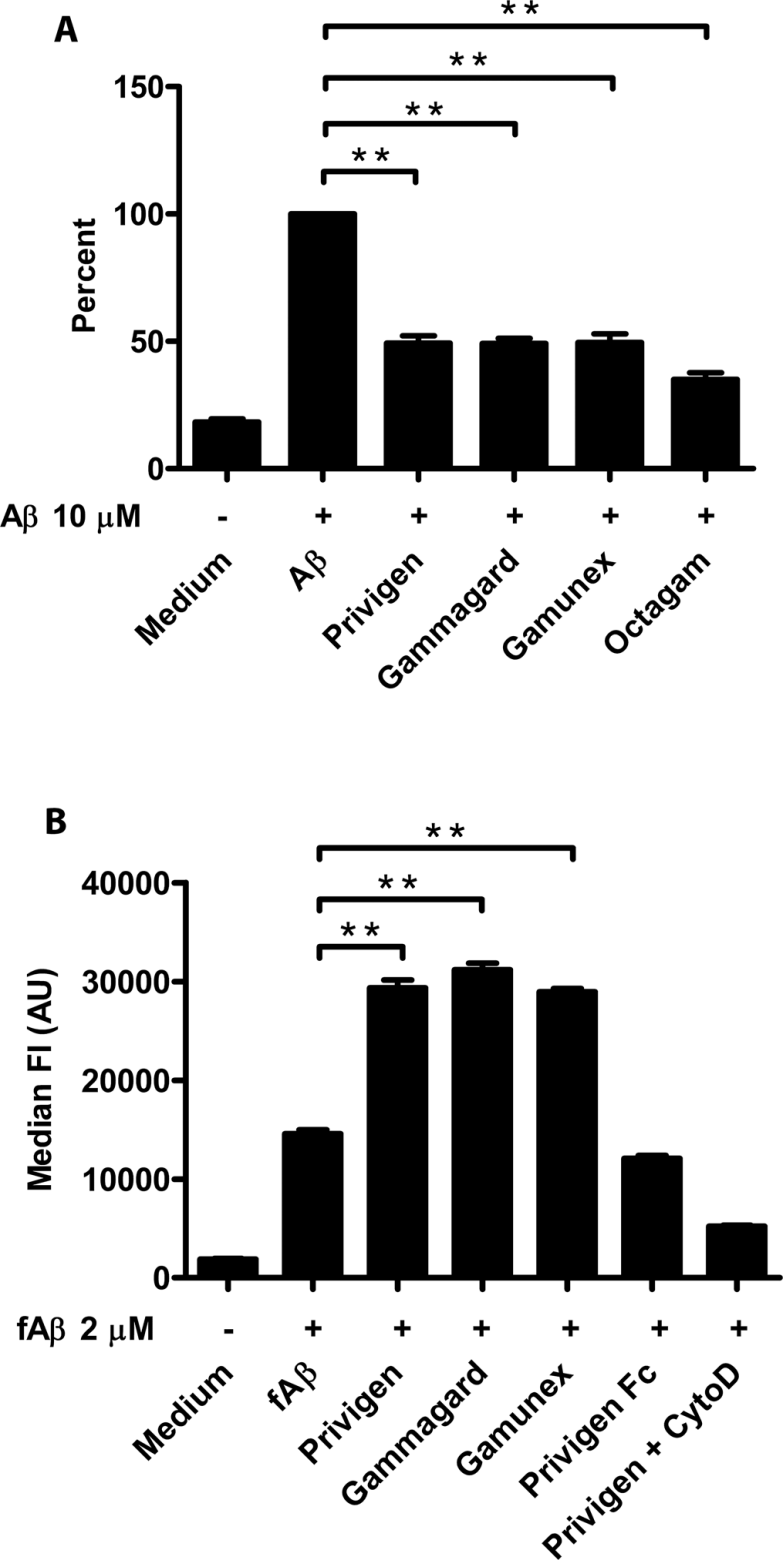
*In vitro* Comparison of commercial IgG preparations activity in toxicity and phagocytosis assay. (A)SH-SY5Y neuroblastoma cells were treated with 10 μM monomeric Aβ42 alone or co-incubated with 100 μM Privigen, Gammagard, Gamunex or Octagam for 72h. Neurotoxicity was determined by LDH assay (**p<0.005). (B) For phagocytosis assay, BV‐2 cells were treated with 2 μM FITC‐labeled Aβ fibrils alone or co‐incubated with Privigen, Gammagard, Gamunex and Privigen Fc at 45 μM, or Privigen 45 μM + Cytochalasin D (**p <0.005). Privigen Fc fragments and Privigen 45 μM + Cytochalasin D were used as negative controls. The panel shows combined results from repeated measurements (n = 3).

We first evaluated the effects on Aβ42-mediated cytotoxicity (Fig 4A). All tested IgG products significantly inhibited Aβ42-induced cytotoxicity in SH-SY5Y cells (**p<0.005) with no significant differences between IgG products. In the phagocytosis assay, BV‐2 cells co‐incubated with 2 μM FITC‐labeled Aβ fibrils with Privigen, Gammagard and Gamunex significantly increased the amount of phagocytosed fibrillar Aβ in the FITC‐positive cell population with no significant differences between products (Fig 4B) (**p<0.005). Also, the negative controls Privigen Fc fragment and Privigen + Cytochalasin D did not increase the uptake of fibrillar Aβ.

These results indicate that the different IgG formulations have an impact on *in vitro* binding properties of low affinity antibodies but do not influence their activity in functional assays. To further investigate the relevance of the observed effects of *in vitro* binding assays in another relevant scenario, we performed an *in vivo* study in rats.

### Formulation-dependent differences in *in vitro* binding have no impact on *in vivo* efficacy

**Fig 5:**
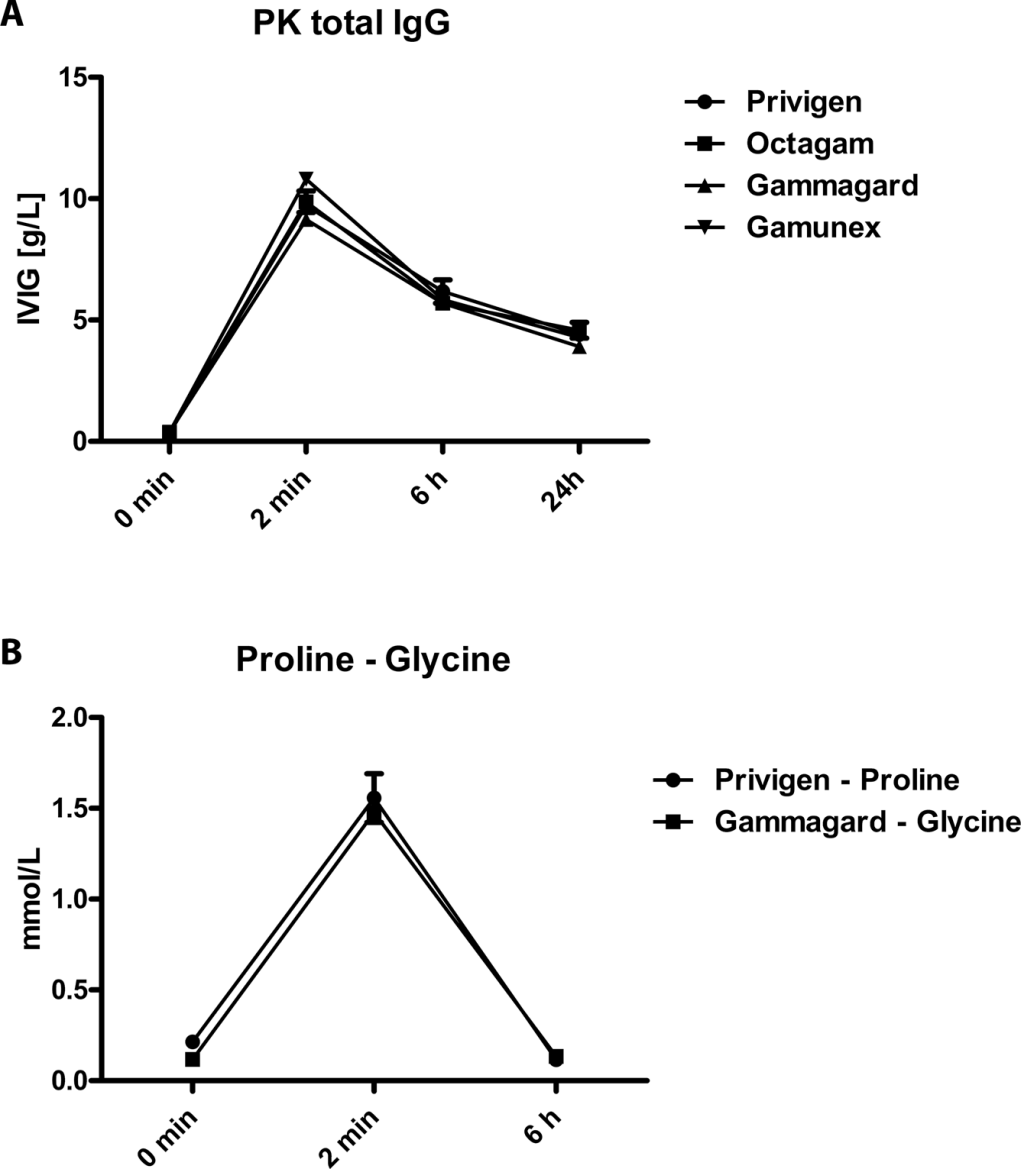
*In vivo* pharmacokinetics of different IgG preparations, proline and glycine in rats. Clr:CD(SD) rats (n = 10 in groups 0 min and 2 min; n = 5 in groups 6h and 24h) were intravenously injected with 500 mg/kg of Privigen 10%, Gammagard 10%, Octagam 10% or Gamunex 10%, repectively. Blood was taken at baseline (0 min) as well as 2 min, 6h and 24h after injection. Plasma (10% citrate) was prepared and analyzed for total IgG by Nephelometry and Proline and Glycine concentration by HPLC. The elimination profile for IgG is shown in (A) and for proline and glycine in (B).

**Fig 6:**
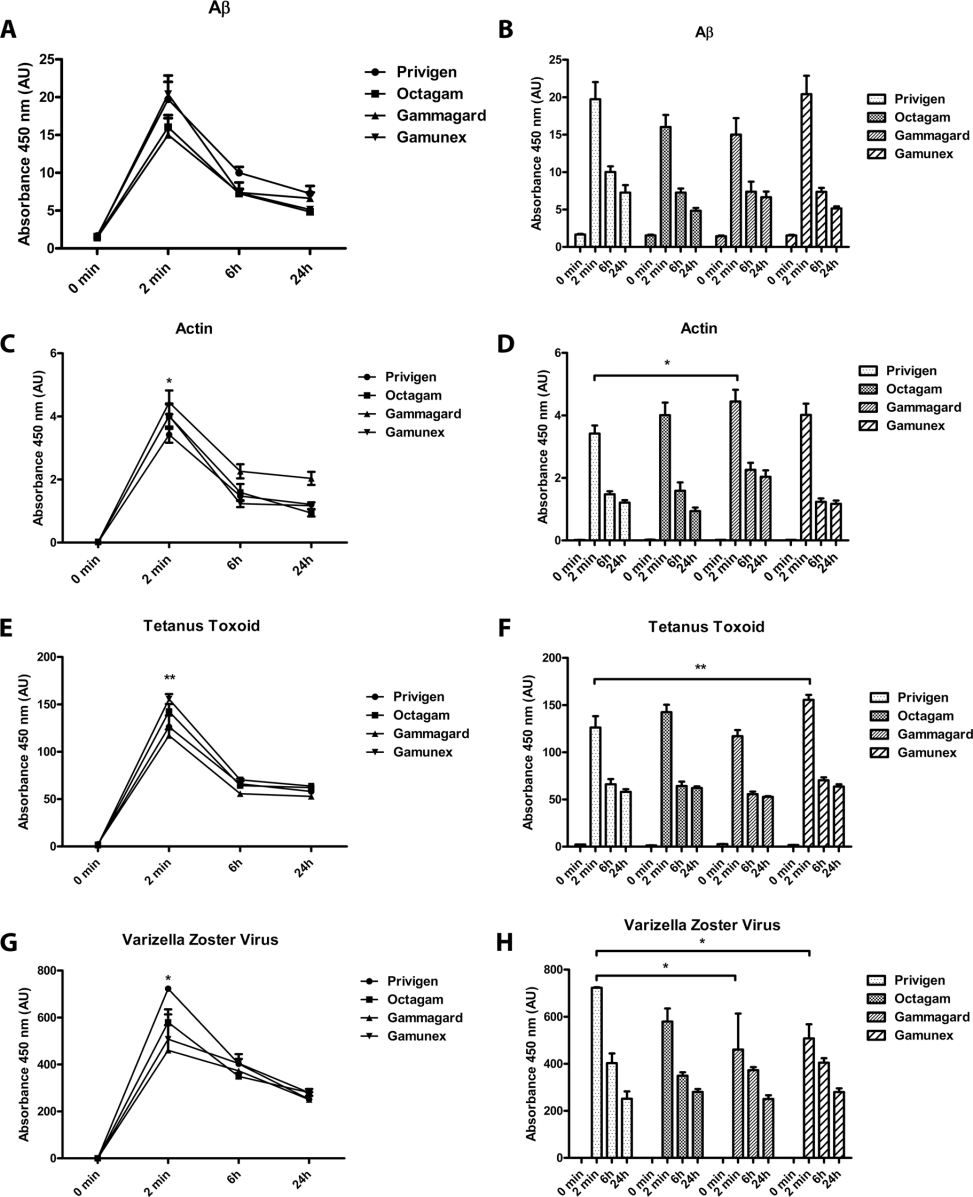
*In vivo* comparison of the binding to Aβ, Actin, tetanus toxoid and Varicella Zoster Virus by ELISA. Clr:CD(SD) rats (n = 10 in groups 0 min and 2 min; n = 5 in groups 6h and 24h) were intravenously injected with 500 mg/kg of Privigen 10%, Gammagard 10%, Octagam 10% or Gamunex 10%, respectively. Blood was taken at baseline (0 min) as well as 2 min, 6h and 24h after injection. Plasma (10% citrate) was prepared and the binding capacity to oligomeric Aβ (A and B), actin (C and D), Tetanus Toxoid (E and F), and VZV (G and H) was analyzed by ELISA. (*p<0.05, **p<0.01). Each ELISA was performed 3 times n = 3 (with all animals n = 30) except VZV t = 2 min n = 2 (with all animals n = 30) due to limited sample volume.

It is assumed that *in vivo* the matrix e.g. stabilizing excipients are quickly diluted, as the half-life of proline/glycine is very short (approximately 6 minutes) [26], compared to the half-life of IgG (approximately 3 weeks) [27]. An animal study was performed in which rats were injected intravenously with 500 mg/kg Privigen, Gammagard, Gamunex or Octagam, respectively. Blood was taken at 0 min, 2 min, 6h and 24h after injection. Analysis of total human IgG levels in the serum samples confirmed published results from human patients [27] with the characteristic elimination profile of IgG, with no differences between the tested IgG preparations (Fig 5A). Analysis of Proline/Glycine levels in the plasma samples revealed that 2 minutes after injection there was still some exogenous Proline/Glycine left in the circulation, whereas after 6 h and later, baseline levels of Proline/Glycine were reached (Fig 5B). Further analysis of the plasma samples revealed that for all tested antigens (Aβ, actin, tetanus toxoid and Varicella Zoster Virus) the elimination rate over 24h *in vivo* was comparable for all tested IgG products (Fig 6A, 6C, 6E and 6G). Furthermore, we found that after 6h there were no significant differences in binding to Aβ42 (Fig 6A and 6B), actin (Fig 6C and 6D), tetanus toxoid (Fig 6E and 6F) and VZV (Fig 6G and 6H) detectable between the different IgG products, whereas after 2 min, still some small but significant differences were detectable between Privigen and Gammagard in the binding to actin (Privigen lower, Fig 6D) and VZV (Privigen higher, Fig 6H). There was also a significant difference between Privigen and Gamunex in the binding to tetanus toxoid (Privigen lower, Fig 6F) at 2 min post injection.

These results show that the differences in binding to endogenous (low affinity) antigens such as Aβ42 and actin *in vitro* do not translate into the same differences in functional assays as well as when analyzing *in vivo* samples.

## Discussion

Intravenous Immunoglobulins have been safely used for more than 30 years for the treatment of a variety of auto-immune and inflammatory diseases due to their anti-inflammatory and immunomodulatory properties [8, 28]. The human immunoglobulin G (IgG) repertoire and therefore also IgG prepared from pooled human plasma, contains endogenous antibodies against the Aβ peptide as well as other self-antigens that arise in the absence of vaccination or passive immunization [2]. For actin, a cytoskeletal intracellular protein, self-reactivity in human serum IgG and therefore plasma-derived IgG preparations has already been reported [29]. These proteins can be taken as representative targets of natural (auto)-antibodies (Nabs) that arise in the absence of an exogenous inflammatory stimulus and therefore have not undergone sequential rounds of affinity maturation. For this reason Nabs are mostly low- to medium-affinity antibodies with a role in immune homeostasis, rather than host defense [2]. High titer antibodies to tetanus toxoid are also present in normal human serum and therefore in IgG preparations, since in general, plasma donors are vaccinated against the tetanus toxin.

In the present study we found that ELISA measurements indicated differences in binding to endogenous low-affinity antigens, such as Aβ and actin between different commercially available IgG preparations but not to tetanus toxoid, an exo-antigen generating high affinity antibodies. To investigate in more detail the possible cause for the observed differences in binding to Aβ and actin between different IgG preparations we used improved ELISA protocols [15] as well as other assays to complement ELISA measurements, which confirmed differential activity against self-antigens between the tested IgG preparations. Other studies [11, 13] found comparable differences between commercially available IgG preparations. For example, Balakrishnan et al found differences in binding to Aβ40 and Aβ42 between different tested IVIG products, which were also analysed in respect to the influence of pH, dimer formation and IgG subclass composition and concluded that the differences in these products can be attributed primarily to the manufacturers' production strategies and storage conditions. Klaver et al. also found differences between three tested IVIG products in the unfractionated and the purified anti-Aβ antibody eluates. They concluded that measurement of these affinity purified antibody eluates may be useful to verify the presence of specific anti-Aβ antibodies because interference of polyvalent antibody binding was less than with unfractionated IVIG. Their observation with unfractionated IVIG products closely mirrors our own results without employing further methods to identify the cause for the differences. Therefore, to better understand the reason for the observed differences in binding assays, we also tested different IgG preparations in cell-based functional assays and found that the differences between the preparations were absent in Toxicity and Phagocytosis assays. To investigate if this effect might be matrix dependent, we analysed one IgG preparation in different formulations of Proline pH 4.8 (Privigen), Glycine pH 4.7 (Gammagard) and Glycine pH 4.2 (Gamunex), respectively. The fact that the same differences in ELISA and real-time binding, e.g. Proline pH 4.8 formulated IgG showed less binding to the self-antigens Aβ and actin than lower pH/Glycine-formulated IgGs, were still present indicates that the *in vitro* binding signals depend on the matrix and not on the antibody composition in the IgG preparation. Also, the fact that binding to Aβ and actin is influenced in a similar fashion shows that the form of Aβ used in the assay is negligible. Different formulations appear to influence binding of low affinity antibodies to self-antigens, which is mainly evident in *in vitro* binding assays but not in functional assays. However, formulations in particular the choice of the stabilizer are mainly designed to improve stability of the IgG preparation [30] and therefore, in doing so may affect the binding particularly of low affinity antibodies to antigens more pronounced in *in vitro* binding assays compared to functional assays. This might be explained by the different mechanism of action occurring in these assays. Binding to antigens in *in vitro* binding assays is solely dependent on Fab function, whereas binding and internalization in functional assays, such as Phagocytosis, is dependent on both, Fab as well as Fc function. Binding of the Fc portion to cell surface receptors might be less influenced by the matrix than binding of the antigen by the Fab portion. Importantly, the matrix effects observed *in vitro* were rapidly lost *in vivo* as demonstrated in our *in vivo* measurements performed on plasma samples taken at different times in the rat study. Exogenous Proline and Glycine were cleared quickly from the circulation and only small differences between the tested IgG preparations in binding to self-antigens after 2 min injection time (i.e. when exogenous glycine or Proline was still present in the circulation) were detected, which became minor after the formulation was cleared from the circulation (6h and 24h).

Therefore we conclude that differences between commercial IgG preparations in binding to endogenous (low affinity) antigens such as Aβ42 and actin are artefactual and only evident in *in vitro* binding assays, whereas in functional assays and *in vivo* measurements different IgG preparations show comparable activity. Thus, ELISA data alone are not appropriate to analyse and rank the low affinity binding capacity and potential efficacy of IgG preparations. Additional methods should be adopted to complement ELISA data.
